# Circular of the Committee Appointed by the Am. Med. Association to Report on the Action of Water on Lead Pipe

**Published:** 1852-02

**Authors:** 


*Circular of the Committee appointed by the Am. Med. Association to report on the Action of Water on Lead Pipe, etc.*

We insert, with pleasure, the following Circular, which, we trust, will call out practical observations on the important subjects assigned to the Committee: —

*To the Members of the Medical Profession in the United States.* — The undersigned, a Committee of the American Medical Association to report on "the action of water on lead pipes, and the diseases which proceed from it," are desirous of obtaining from their professional brethren any information that is calculated to throw light on this important, but hitherto generally unobserved subject. They therefore take the liberty of proposing the following questions: —

1st. Have you, in your practice, met with cases of lead or painter's colic produced by using water drawn through lead pipes, or contained in leaden cisterns?

2d. Have you met with cases of arthralgy? If so, have they been attributable to this cause?

3d. Have painful neuralgic diseases been observed by you, among persons using water thus exposed to lead?

4th. Have you seen instances of lead encephalopathy?

5th. Have you observed paralysis as a precursor, concomitant or sequel to either of the above forms of disease?

Answers to any or all of the foregoing questions, and any facts or information as to any form of disease originating in the use of water impregnated with lead, will be very gratefully received. Accurate descriptions of all cases would be very desirable, especially their early history. It will also be very important to know the length of time each individual case had been exposed to lead before the disease became manifest.

As the report must be made at the annual meeting of the Association to be held in Richmond, Va., in mny next, it is desirable that all information should be forwarded to any one of the Committee previous to the first of March next.

HORATIO ADAMS, Waltham Mass.,

SAM'L L. DANA, Lowell, "

JOHN C. DALTON, " "

} *Committee.*

Waltham, Dec. 5th, 1851.

